# Label Self-Advised Support Vector Machine (LSA-SVM)—Automated Classification of Foot Drop Rehabilitation Case Study

**DOI:** 10.3390/bios9040114

**Published:** 2019-09-27

**Authors:** Sahar Adil Abboud, Saba Al-Wais, Salma Hameedi Abdullah, Fady Alnajjar, Adel Al-Jumaily

**Affiliations:** 1 Computer Engineering Department, University of Technology, Baghdad, Iraq; 120028@uotechnology.edu.iq; 2 Faculty of Engineering and Information Technology, University of Technology, Broadway, Sydney NSW 2007, Australia; 120015@uotechnology.edu.iq; 3 Bio-Medical Engineering Department, University of Technology, Baghdad, Iraq; 60004@uotechnology.edu.iq; 4 Department of Computer Science and Software Engineering, College of Information Technology, UAE University, Al Ain 15551, UAE

**Keywords:** foot drop, Support Vector Machine, electromyography, label classification, rehabilitation devices

## Abstract

Stroke represents a major health problem in our society. One of the effects of stroke is foot drop. Foot drop (FD) is a weakness that occurs in specific muscles in the ankle and foot such as the anterior tibialis, gastrocnemius, plantaris and soleus muscles. Foot flexion and extension are normally generated by lower motor neurons (LMN). The affected muscles impact the ankle and foot in both downward and upward motions. One possible solution for FD is to investigate the movement based on the bio signal (myoelectric signal) of the muscles. Bio signal control systems like electromyography (EMG) are used for rehabilitation devices that include foot drop. One of these systems is function electrical stimulation (FES). This paper proposes new methods and algorithms to develop the performance of myoelectric pattern recognition (M-PR), to improve automated rehabilitation devices, to test these methodologies in offline and real-time experimental datasets. Label classifying is a predictive data mining application with multiple applications in the world, including automatic labeling of resources such as videos, music, images and texts. We combine the label classification method with the self-advised support vector machine (SA-SVM) to create an adapted and altered label classification method, named the label self-advised support vector machine (LSA-SVM). For the experimental data, we collected data from foot drop patients using the sEMG device, in the Metro Rehabilitation Hospital in Sydney, Australia using Ethical Approval (UTS HREC NO. ETH15-0152). The experimental results for the EMG dataset and benchmark datasets exhibit its benefits. Furthermore, the experimental results on UCI datasets indicate that LSA-SVM achieves the best performance when working together with SA-SVM and SVM. This paper describes the state-of-the-art procedures for M-PR and studies all the conceivable structures.

## 1. Introduction

Stroke represents a major health problem in today’s society. One of the consequences of stroke is foot drop. Foot drop is a weakness that appears in specific muscles in the ankle and foot such as: the anterior tibialis (AT), gastrocnemius (Gas.), plantaris and soleus muscles. Foot flexion and extension are normally generated by lower motor neuron (LMN). Lesions in the lower motor neuron (LMN) will cause foot drop. Foot drop manifests in 52% to 67% of patients with spinal upper motor neuron (UMN) [1]. Foot Drop (FD) is a common disorder without specifying to age and affects around 1% of women and 2.8% of men [2]. Bio signal control systems like electromyography (EMG) are used devices that target leg rehabilitation. These devices target various leg impairments, including foot drop. Electromyography (EMG), which records myoelectric signals from muscle activity, has been widely used to detect the user’s intended action [3]. The EMG electrodes are placed in the subject’s limb, either in an invasive or non-invasive way. A wide range of people prefer not to implant electrodes inside the body and instead prefer to use a surface EMG (sEMG). However, the sEMG has numerous disadvantages such as crosstalk from other muscles and robustness. Furthermore, it is hard to acquire a myoelectric signal from deeper muscles, so it is difficult to deal with myoelectric signal processing using EMG. Myoelectric pattern recognition (M-PR) methods can also be applied to artificial intelligent applications. This system contains multiple steps—first the sEMG data is filtered from the noisy signal, then it is applied to extract and reduce features, to minimize the large sample data. In addition, methodologies such as support vector machine (SVM), cSA-SVM, vSA-SVM and label classifications are studied in this paper. One of the popular machine learning methodologies is the support vector machine (SVM) method, which is used to classify data. Vapnik [3,4] proposed the support vector machine as an influential classification method, various forms of SVM were presented in the literature and applied in several different applications. SVM categorizes class problems into two; classes and multi-classes. The SVM method gives an ideal decision for the formation of a boundary between two or multiple classes. The margin created, separates the classes and the decision boundary is maximized. To theorize the binary classification by a training set of N samples, a vector for the input data should be considered, which depends on the ith sample. This is labeled related to its class. The purpose of SVM is to separate the binary labeled training data within the hyperplane that has a maximum distance from them. This is called a maximum margin hyperplane [3,5]. The standard SVM disregards the train data that is not separated linearly by the kernels through the training stage, which occurs during the outline of the tolerance parameters in the impartial function and restrictions. For this reason, it will be classified incorrectly if the data are similar or match the misclassified data that appears in the test set. This happens when the data that are close to the misclassified data are unspecified. This results in a misclassification that is not sensible and not controlled [6]. A non-repeating, self-advising method for SVM was adapted [7], which extracts consequent knowledge from the training phase without the addition of extra parameters. The misclassified data is supplied from two prospective sources. First, from outliers and second, from the data that has not been separated correctly [8]. Many researchers have adapted versions of SVM with the goal of raising the classification efficiency and performance for some applications. Use of label classification on experienced knowledge is one of the methods that has been used without increasing the cost since there is no addition of an extra parameter. For example, they proposed novel methodologies for texture analysis to improve the single-label classification of facial features [9,10]. Masood, A. et al. [6] suggested to enhance label classification with class classification techniques. In addition, they dealt with the problem of limited labeled data available, especially for histopathological images. They proposed a novel learning model, created on a deep belief neural network and semi-advised SVM to make effective use of labeled data along with unlabeled data for the training phase. It displayed improved performance when matched with different state-of-the-art approaches for skin cancer diagnosis. The proposed model was used for diagnosing skin cancer. Multi-label classifying is a predictive data mining application with multiple applications in the world, including automatic labeling of resources, including videos, music, images and texts. The multi-label data can be used as a learning tool and can be achieved by different methods, such as: the problem transformation method which has two common methods, the adaptation method, and ensembles of classifiers [3]. Several applications include research work with the use of label classification methods (LCM) to improve the progression of the search for related information on Twitter. Five different labels are defined to categorize tweets, including news. The system was proposed to analyze complex motion in events; it combines the tracking and multilabel hypergraphs of moving targets in video sequences [11]. The adaptation methodologies state that some classification models were primarily intended for resolving binary problems and then were expanded to solve multi-class problems. By contrast, other methods can easily work with several classes. In this case study, the novel recognition system, that is an integration of label classification methods with SA-SVM is used to get LSA-SVM for two-class classification, to overcome the problems and improve the reliability of the diagnosis process. It is important to develop computational tools for automated diagnosis that operates on a quantitative measure. Such tools can facilitate objective mathematical judgment complementary to that of medical experts and help them to identify the affected areas more efficiently with more accurate diagnosis and less wastage of time in treatment while trying not to lose the bounder for working in real time standard.

## 2. Related Work

### 2.1. The Standard SVM

The basic idea of the SVM, which simplifies the pair (w,b) is described in the hyperplane with the equation <w,x>+b=0. SVM can utilize to produce a non-linear decision function, by projecting the training data to a higher dimensional internal product space, known as feature space, by applying a non-linear map $\phi \left(x\right):R^{n}\to R^{d}$.

Although the optimal linear hyper-plane calculates in the feature space, by applying kernels it is capable to make necessary processes in the input space using $k\left(x_{i},x_{j}\right)\;=\;<\phi \left(x_{i}\right),\phi \left(x_{j}\right)>$ which is an internal product in the feature space. In terms of these kernels, the decision function can be written as the following Equation (1):

(1) $$f\left(x\right)=sign(\Sigma_{\alpha_{i}>0}\;y_{i}\alpha_{i}k\left(x,x_{i}\right)+b)$$

The decision value for each *X* of the test sets have either a negative or a positive value, depends on the situation of *X* and the hyperplane that has been clarified as Equation (2):

(2) $$h\left(x\right)=\Sigma_{\alpha_{i}>0}\;y_{i}\alpha_{i}k\left(x,x_{i}\right)+b$$

There are three mutual kernel functions in SVM—Radial Basis Function kernel (RBF), Polynomial kernel and Sigmoid kernel. This paper has used the RBF kernel as Equation (3):

(3) $$k\left(x_{i},x_{j}\right)=e^{-\gamma |x_{i}-x_{j}{|}^{2}}$$

### 2.2. Self-Advised Support Vector Machine (SA-SVM)

Advised Weighted-SVM treats the neglect of SVM from the information that can be obtained from misclassified data. By the creation of advised weights, which depends on the distance between the misclassified train data and the classified train data. In addition, applying these weights together with decision values of SVM in the test phase, assist the procedure to reduce the outlier data [12,13]. The details of the Self-Advised SVM procedure is demonstrated in the following steps:

1. Classify the hyper-plane founded by applying the decision function is Equation (4):
  (4) $$f\left(x\right)=sign(\Sigma_{\alpha_{I}>0}\;y_{i}\alpha_{i}k\left(x,x_{i}\right)+b)$$
2. Misclassified data that samples in the first train phase are recognized. The misclassified datasets (MD) in the training phase are calculated by Equation (5):
  (5) $$MD=\cup_{i=1}^{N}X_{i}|y_{i}\neq sign\left(\Sigma_{\alpha_{j}>0}\;y_{i}\alpha_{j}k\left(x,x_{j}\right)+b\right)$$
  The MD set may be null but the empirical outcomes appear when the presence of misclassified data in the training phase is a communal existence. It should be recognized that trying any technique to benefit from misclassified data should have a control to affect the outlier data. When the misclassified data is included to resemble samples, the use of misclassified data improved the classification accuracy [14].
3. The algorithm indicates: If MD is null then go to the testing phase or else compute neighborhood length (NL) for each Xi of MD. Equation (6), defined NL.
  (6) $$NL\left(x_{i}\right)=minimum_{x_{i}}\left(\right\|x_{i}-x_{j}\left\|\right|y_{i}\neq y_{j})$$
  where $X_{j}=1,\cdots ,N$ is the training data that does not belong to the MD set. If the training data is a map with a higher dimension, the distance between xi and xj can be evaluated in Equation (7) with reference to the related RBF kernel
  (7) $$\|\theta \left(x_{i}\right)-\theta \left(x_{j}\right)\|={\left(k\left(x_{i},x_{i}\right)+k\left(x_{j},x_{j}\right)-2k\left(x_{i},x_{j}\right)\right)}^{0.5}$$
4. Calculating Advised Weight AW(xk) for each sample xk from the test set using Equation (8). These AWs represent the closest test data to the misclassified data.
  (8) $$\left\{\begin{array}{c} 0\;\;\;\;\;\forall x_{i}\in MD,\left\|x_{k}-x_{i}\right\|>NL\left(x_{i}\right)orMD=NU,\\ \Sigma 1-\frac{\Sigma_{x_{i}}\left\|x_{k}-x_{i}\right\|}{\Sigma_{x_{i}}NL\left(x_{i}\right)}\;\;\;\;\;x_{i}\in MD,\left\|x_{k}-x_{i}\right\|\leq NL\left(x_{i}\right) \end{array}\right.$$
5. The absolute value of the SVM decision values for each xk from the test set are considered and scaled in to [0, 1].
6. Finally, for each xk from the test set in Equation (9):
  If AW(xk)< decision value (xk) then:
  (9) $$y_{k}=sign\left(\Sigma_{\alpha_{j}>0}y_{j}\alpha_{j}k\left(x_{k},x_{j}\right)+b\right)$$
  which is identified with normal SVM, otherwise:
  $$y_{k}=y_{i}\left|(\right\|x_{k}-x_{j}\left\|\left(x_{i}\right),x_{i}\in MD\right)$$

### 2.3. Label Classification

Label Classification (LC) contains two types of classifying—Single Label Classification (SLC) and Multi-Label Classification (MLC)—which are the supervised learning problems where sample data are connected to single or multiple labels. Applications that use the SLC and MLC have increased in different fields. For example, text classification, scene and video classification, bioinformatics and Biomedical Text Data [15]. Generally, Binary Relevance (BR) is a method used for Multi-Label Classification (MLC). Label Classification LC deliberates every label as an independent binary problem and its work depends on the lack of appearance for the non-direct modeling label correlations. Most of the existing methods contribute to complexity of model inter-dependencies between the labels. Another method used in Multi-Label Classification is to perform problem transformation, where a multi-label problem transformed into one or more single-labels like binary or multi-class problems. That activates single-label classifiers and is transformed by their single-label predictions into multi-label predictions. Problem transformation is used to describe both flexibility and scalability. They apply Support Vector Machines, Naive Bayes, k Nearest Neighbor methods and Perceptron [16]. Where $X^{d}\subset R$ the input sample domain, the sample feature formed as a vector *d*. The sample input forms a vector of d feature $X=[x_{1},\cdots ,x_{d}]$, while the *l* output domain is L=1,⋯,L. Each sample (x) related to a subset of these labels, which forms as L vector $y=[y_{1},\cdots ,y_{L}]$. Where $y_{1}=1$, if and only if label *j* is related with sample *x* and 0 otherwise. They assume a set of training data *D* of *N* labeled patterns as $D=(x^{i},y^{i})|i=1,\cdots ,N$. So that the researcher can write multi-label accuracy equation for a set of *N* test as follow in Equation (10):

(10) $$Accuracy=\frac{1}{N}\Sigma_{i=1}^{N}\frac{|y^{i}\wedge {\hat{y}}^{i}|}{|y^{i}\vee {\hat{y}}^{i}|}$$

Ensemble Classifier Chains (ECC) signifies a vector of absolute outputs $\hat{W}=\left[{\hat{w}}_{1},\cdots ,{\hat{w}}_{L}\right]\in R^{L}$, while ${\hat{W}}_{j}$ signifying the absolute for the $j_{th}$ label. For prediction vectors ${\hat{y}}_{1},\cdots ,{\hat{y}}_{m}$ from repeating 1,⋯,m, The absolute equation evaluate as in Equation (11):

(11) $${\hat{W}}_{j}=\frac{1}{m}\Sigma_{k=1}^{m}{\hat{y}}^{i}k$$

Also, threshold function f apply ${\hat{w}}_{j}$ to get a bipartition of appropriate and inappropriate labels: $\hat{y}=f_{t}\left(\hat{w}\right)$ Softmax functions also offer single-label classification. Practically, it applies to the multi-label scenario by problem transformation. Softmax loss function can modify the multilabel scenario as shown in Equation (12) [17]:

(12) $$l_{softmax}=\Sigma_{y\in Y_{i}}\log\left(\frac{\exp(f_{y}\left(x_{i}\right))}{\Sigma_{j\in y}xp\left(f_{y}\left(x_{i}\right)\right)}\right)$$

## 3. Materials and Methods

### 3.1. Materials

**Figure 1:**
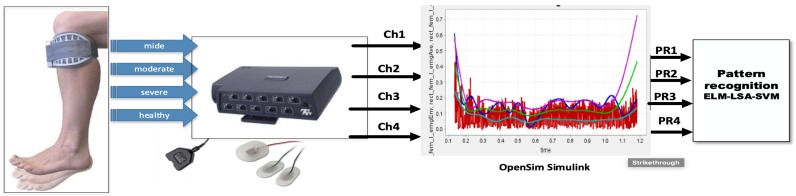
Stages for an Offline myoelectric pattern recognition system.

**Figure 2:**
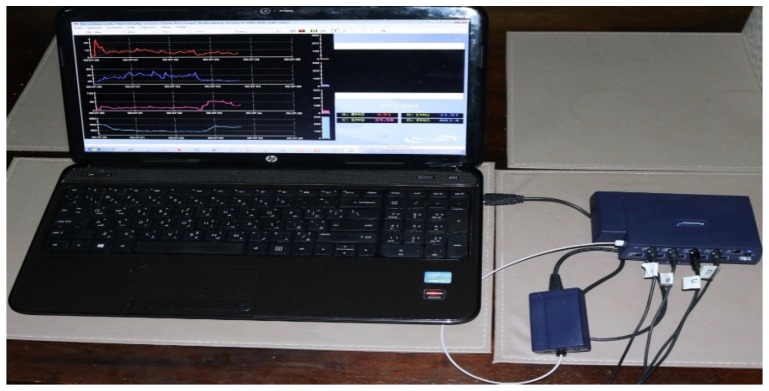
The hardware and software needed for the off-line application.

The Myoelectric Pattern Recognition system consists of two main parts—the software and hardware that are further elaborated in the phases of the real-time M-PR system, as visible in Figure 1 and Figure 2. Overall, all phases of real-time pattern recognition based sEMG is displayed in Table 1. For the experimental stages, the system collects the data from healthy and unhealthy subjects. The collected data is then applied to train the system, resulting in the output of classification; the trained classifier, the OpenSim prediction and simulation for gait level.

The collected EMG signals were processed on a Personal Computer Intel Core i7, 2.8 GHz with 16 GB RAM and equipped with a Windows 10 operating system. A band-pass filter was used for filtering the signals in the frequency band (25–550 Hz). A notch filter was applied to remove the 50 Hz line nosiness. The EMG signals were downsized to 1000 Hz to minimize the size. To estimate the recommended mSA-SVM, LSA-SVM and ELM-LSA-SVM, the experiment embraced 13 datasets from the UCI machine learning repository [18,19] These databases were nominated from the most public benchmarks for classification and diagnosis. The diversity of these databases supports the authentication in this study. The number of instances and the attributes of each database shown in the tables in each chapter was used. It should be noted that for a dataset with multi-class we used 6 datasets while for two classes 7 datasets [20].

### 3.2. Procedure for Collecting sEMG Signal Data

**Figure 3:**
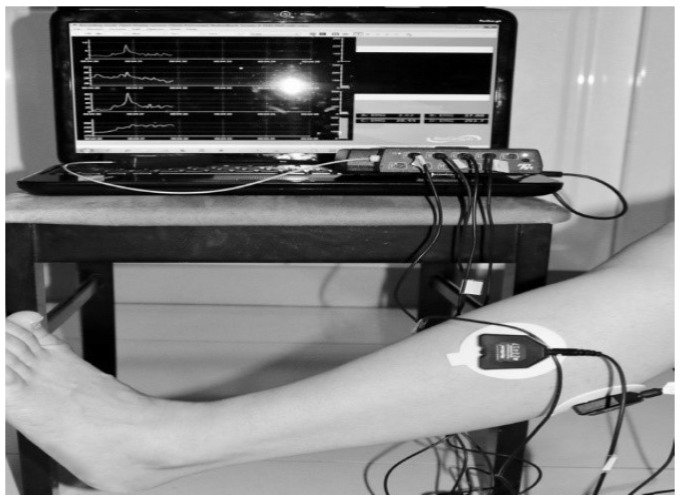
Knee Flexion with Flexion Dorsiflexion.

The data wre collected from the hospital, based on the design procedure that received ethical approval to collect data from FD patients in Metro Rehabilitation Hospital in Sydney, Australia using Ethical Approval (UTS HREC NO. ETH15-0152). For the experiment, data were collected from 13 subjects. These 13 subjects involved in the offline experiment; consisted of 6 females and 7 males, aged between 18 to 84 years; the average age being 51 years. Ten of them were affected with Foot Drop. The other three were healthy with no muscle disorder. During the experiment the subjects were seated, so that the knee is in a fixed position as shown in Figure 3, to avoid the influence of position movements on EMG signals. A few digital filters were applied during data collection. The filters applied were a band pass filter between 25 and 550 Hz and a notch filter to remove the 50 Hz line noise. The EMG signals were reduced to 1080 Hz.

**Figure 4:**
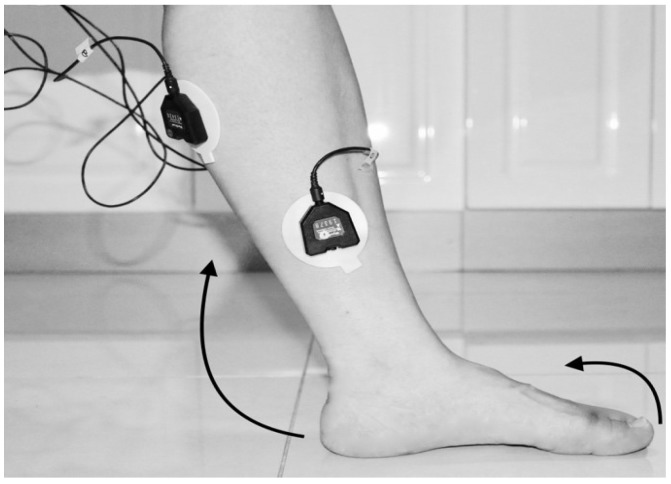
Collect data signals from the leg using sEMG device.

**Figure 5:**
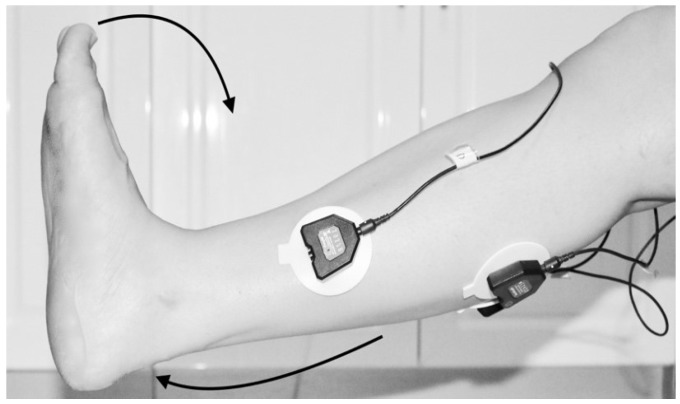
Knee Extension with Flexion Dorsiflexion.

**Figure 6:**
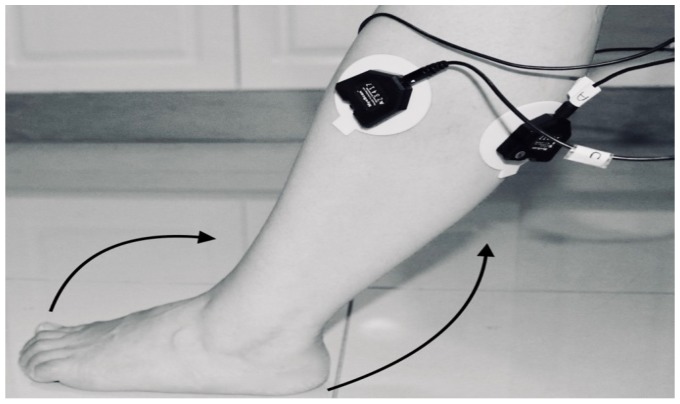
Knee Flexion with Flexion Dorsiflexion.

**Figure 7:**
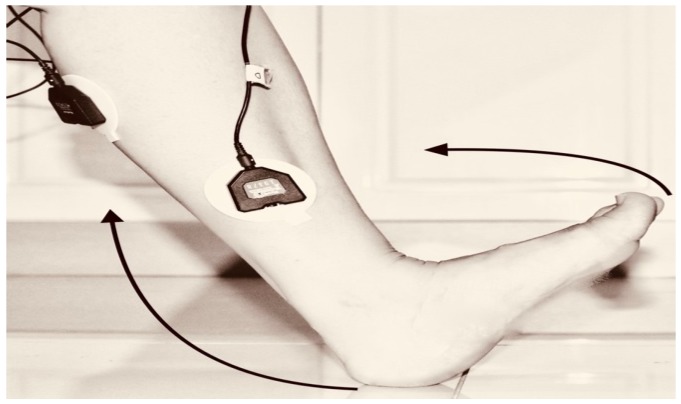
Knee Flexion with Flexion Dorsiflexion.

**Figure 8:**
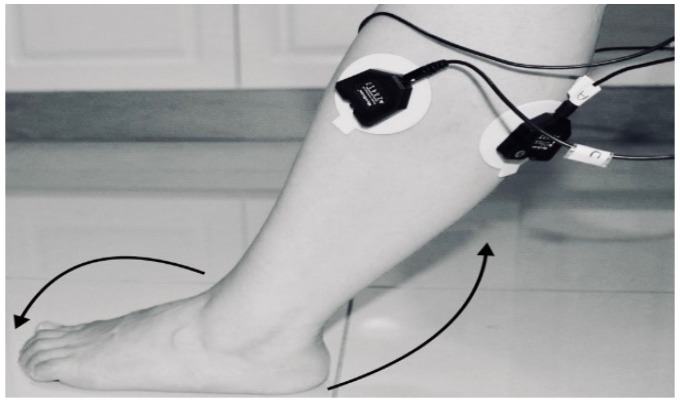
Knee Flexion with Extension Planter Dorsiflexion.

**Figure 9:**
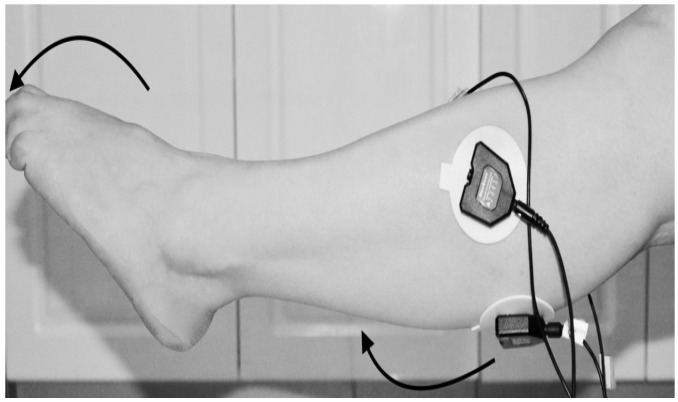
Knee Extension with Extension Plantarflexion.

The classification was dependent on the sample data from which it was acquired. The data were collected under the Supervision of the Stroke Coordinator at the Metro-Rehab Hospital. The data was collected in three phases at each posture (flexion or extension) of the knee joint. The healthy and unhealthy subjects, went through the following:

**First trial:** To move (with help, if required) his/her lower limb at the knee joint. Flexion and extension (bend and straighten) from the actual position while sitting on a chair. Each set of trials took 3 s and a 5 s rest was given between two trials (two sets of trials were done for this case) as shown in Figure 4 and Figure 5.
**Second trial:** To move (with help, if required) his/her foot up and down as much as possible from rest position while sitting on the chair. Each set of trials took 3 s and a 5 s rest was given between two trials. (Five sets of trials were done for this case) as shown in Figure 6 and Figure 7.
**Third trial:** To move (with help, if required) his/her lower limb (foot and leg) at the knee joint, to flex or extend (bend and straighten) with Extension Plantar flexion and Flexion Dorsiflexion from the rest position while sitting on the chair. Each set of trials took 3 s and a 5 s rest was given between two trials. (Two sets of trials were done for this case) as shown in Figure 8 and Figure 9.

**Figure 10:**
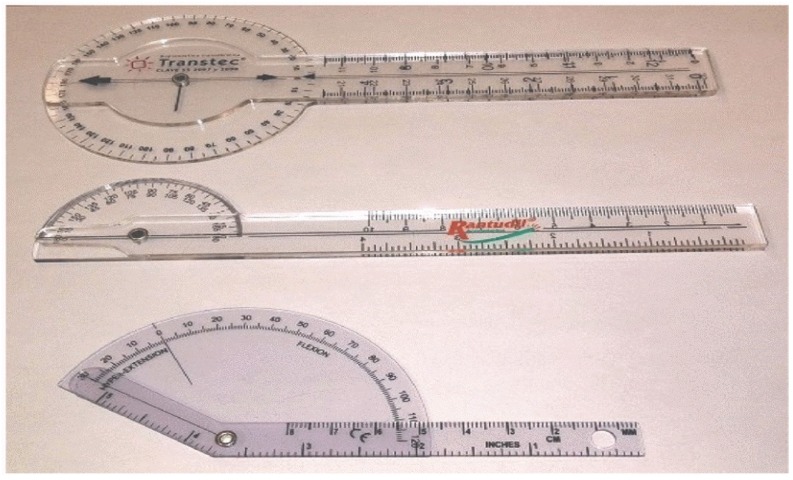
Measure Dorsal Plantar Flexion range for the leg [21].

Data for 12 s was collected for each subject’s trial and data for 156 s were collected for all repetitions by the subject. Three-fold cross-validation was conducted with the offline classification. To measure the Dorsal Plantar Flexion range for the leg, we used the Goniometric Measurement such as Protractor (Angle Finder and Bevel Square Head) as shown in Figure 10. Table 2 presents the characteristics for sick and healthy subjects collected from Metro Rehabilitation Hospital.

For the Metro-Hospital dataset, we collected the Surface electromyography (EMG) signals from 13 subjects, from Rectus Femurs (RF), Gastrocnemius (Gas), Soule (Sol) and Tibias Anterior (TA). The OpenSim dataset for CG, provides Surface electromyography (EMG) signals which recorded from ten subjects. sEMG collects signals from the Medial Hamstrings (mH), Biceps Femurs long head (BF), Rectus Femurs (RF), Gastrocnemius (Gas) and Tibias Anterior (TA). Each trial was 5 s with 2 repetitions, which makes 10 s in total. Three trials were done for each subject = 30 s 1000 in each class = 30,000 and there were 4 classes, so data = 120,000/channel. There were 4 channels, which makes the entire sample data = 480,000. The data collected were divided into training data and test data using 3-fold cross-validation.

### 3.3. Method: Label Self-Advised Support Vector Machine (LSA-SVM)

The following concepts describe the LSA-SVM, where the misclassified data was based on calculating the neighborhood length using the label of data instead of the value for single classification method. This procedure would minimize the time process and large data can be processed.

**Figure 11:**
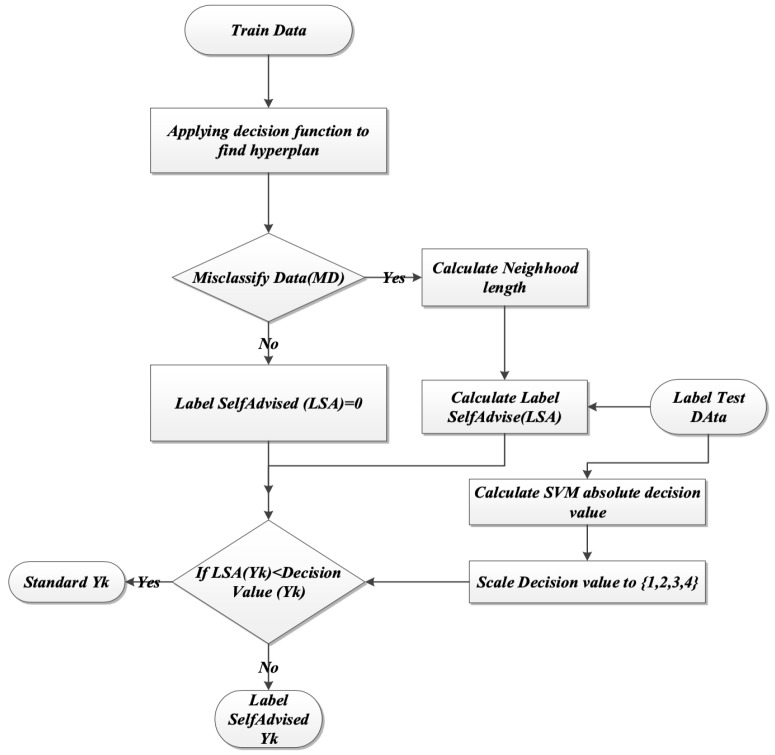
Flowchart of the LSA-SVM methods steps.

1. Applying the decision function as in Equation (10), to classify hyperplane:
  (13) $$f\left(x\right)=sign(\Sigma_{\alpha_{i}>0}y_{i}\alpha_{i}k\left(x,x_{i}\right)+b)$$
  where $x_{i}$ is the input vector for $i_{th}$ sample labeled with $y_{i}$ related to its class, while $\alpha_{i}$ is the non-negative Lagrange multiplier, which conflicts with standard SVM training.
2. Misclassified data samples in the first train phase are recognized. The misclassified data sets (MD) in the training phase are calculated by Equation (11):
  (14) $$MD=\cup_{i=1}^{N}X_{i}|y_{i}\neq sign\left(\Sigma_{\alpha_{j}>0}y_{i}\alpha_{i}k\left(x_{i},x_{j}\right)+b\right)$$
3. The algorithm indicates that: If MD is empty, then go to the testing phase, or else calculate neighborhood length (NL) for each yi of label MD. Equation (12) defined NL.
  (15) $$NL\left(y_{i}\right)=minimum_{y_{i}}\left(\right\|y_{i}-y_{j}\left\|\right|x_{ij})$$
  where $y_{j},j=1,\cdots ,N$ is the label of training data that do not belong to the label of MD set. The label of the training data is mapped to a higher dimension, the distance between $y_{ou}$ and $y_{j}$ is computed according to the following Equations (13) and (14) with reference to the related RBF kernel.
  (16) $$\|\phi \left(y_{i}\right)-\phi \left(y_{j}\right)\|={\left(k\left(y_{i},y_{i}\right)+k\left(y_{j},y_{j}\right)-2kk\left(y_{i},y_{j}\right)\right)}^{0.5}$$
  that is, RBF will be:
  (17) $$k\left(y_{i},y_{j}\right)=e^{-\gamma |y_{i}-y_{j}{|}^{2}}$$
4. For each label yk from test data, the Lab Advised Weight LAW (yk) figures out as Equation (15). These LAWs represent how close the label test data are to the label of misclassified data.
  (18) $$\begin{array}{c} \begin{array}{cc} \; & LAW(yk)=\\ \; & \left\{\begin{array}{c} 0,\;\;\forall x_{i}\in MD,\left\|y_{k}-y_{i}\right\|>NL\left(y_{i}\right)\;orMD=NUL,\\ \Sigma 1-\frac{\Sigma_{y_{i}}\left\|y_{k}-y_{i}\right\|}{\Sigma_{y_{i}}NL\left(y_{i}\right)},\;\;x_{i}\in MD\left\|y_{k}-y_{i}\right\|\leq NL\left(y_{i}\right) \end{array}\right. \end{array} \end{array}$$
  The absolute value of the SVM decision values for each xk from the test set is calculated and scaled to [0,1].
5. For each $y_{k}$ from the label of the test set,
  If $(LAW\left(y_{k}\right)<$ decision value $\left(y_{k}\right)$ then $y_{k}=sign\left(\Sigma_{\alpha_{j}>0}y_{i}\alpha_{j}k\left(x_{k},x_{i}\right)+b\right)$ which is compatible with normal SVM labeling, otherwise $y_{k}=y_{i}\left|(\right\|y_{k}-y_{i}\left\|\leq NL\left(y_{i}\right)\;and\;x_{i}\in MD\right)$. Figure 11, explain the flow chart for the steps above.

## 4. Experiments and Results

**Figure 12:**
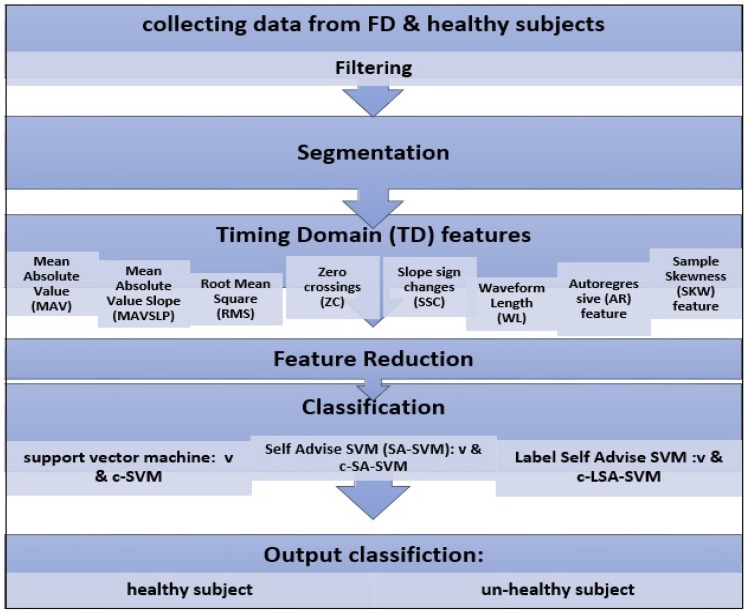
Flowcharts for each stage of M-PR.

The experiments check the performance of LSA-SVM for a single class as conducted through the state of the art pattern recognition system shown in Figure 12. This Flowchart describes each of the methods that were applied to get the output; classifying subjects as healthy and unhealthy using the novel.

### 4.1. Experiments on Hospital Datasets

Various experiments were done to test the performance of LSA-SVM in the Myoelectric pattern recognition. First, we adjusted the parameters of C and g from the range of (2-9, 2-8, …, 29,210), as shown in Table 3, then we examined the performance of accuracy while we apply v-SVM, c-SVM, v-SA-SVM, c-SA-SVM and LSA-SVM. We compare all the classifiers. Some analysis is given in each experiment; in the first experiment we used the first data set FD and the second data set CG to estimate accuracy for each classifier. The second experiment applies the third datasets from UCI.

**Figure 13:**
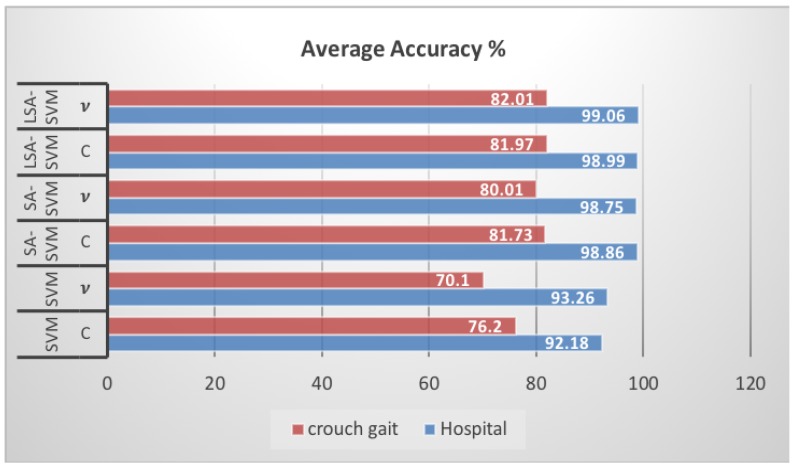
The average classification accuracy of LSA-SVM across two datasets using five-fold cross-validation compared with other classifiers.

Figure 13 and Table 4 specify that the accuracy of LSA-SVM was higher than SA-SVM and other classifiers in all types of dataset. The average accuracy for vLSA-SVM as compared with all other five classifiers is a little higher—equal to 99.06%—while in vSA-SVM it was equal to 98.75% for the FD Hospital dataset. However, for the CG OpenSim dataset vLSA-SVM gives 82.01% and vSA-SVM equal to 80.01% with five-fold cross-validation training.

**Figure 14:**
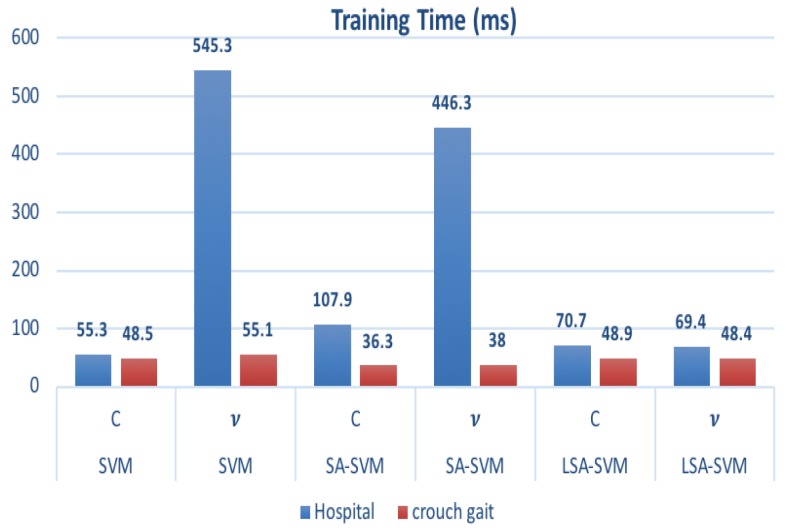
The training time consumption for each type of classifier.

Table 5 and Figure 14 show that the training times of vSA-SVM in two groups of datasets are much faster than those of vLSA-SVM. In that case, the LSA-SVM method did not achieve the best performance, whereas time consumption was equal to 69.4 msec for vLSA-SVM and 46.3 msec, for vSA-SVM but it still reached a real time that is less than 300msec as a standard real time measurement [22,23]. Overall, in most cases, the adaptation of the Label function used in LSA-SVM can improve the performance of the classical support vector machine and SA- SVM.

### 4.2. Experiments on UCI Datasets

LSA-SVM proved that it is capable of classifying four classes of leg movements consisting of a combination of three classes of the unhealthy leg (Mild, Moderate and Severe patients) and the fourth is the healthy class. This section investigates the performance of LSA-SVM on the benchmark dataset that is accessible online on the UCI machine learning website. The experiment depends on the size of the data. We implement 3-fold cross-validation on the larger sized data, while we executed 5-fold cross validation for the small and medium-sized data. Table 6 shows the data specification for a benchmark for 2-Classes dataset types.

This experiment involved six classifiers—v-SVM, c-SVM, c-SA-SVM, v-SA-SVM, c-LSA- SVM, v-LSA-SVM. The optimal parameters are established and noted its effects on the accuracy and time performance of the classifier. Table 7 provides all parameters that were utilized in this experiment.

**Figure 15:**
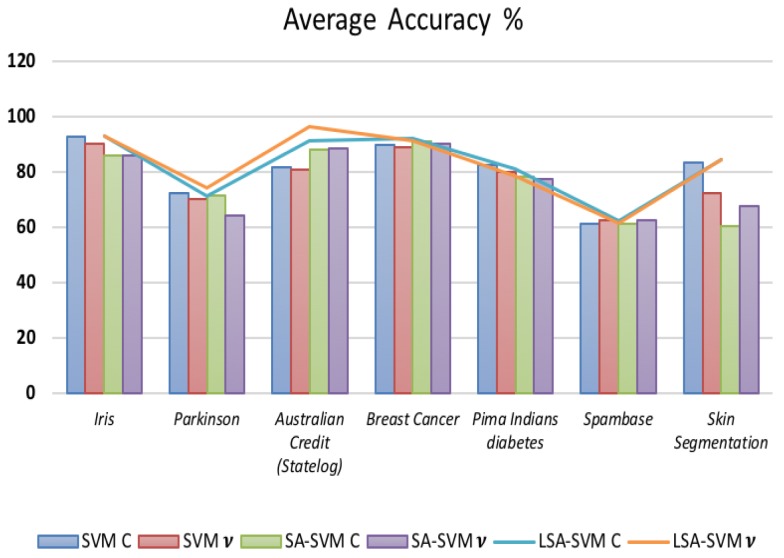
The accuracy of seven classifiers on different data using 5-fold cross validation for small and medium-size data and 3-fold cross validation for large size data for 2 classes.

Table 8 and Figure 15 show that LSA-SVM had performed reasonably through a seven different data sets with 2 classes. The comparison of LSA-SVM and SA-SVM shows that average accuracy are quite similar in some dataset. As for LSA-SVM, the accuracy of v- LSA-SVM is significantly better than c-LSA-SVM only in “Breast Cancer” equal to 92.09 dataset. From the results, recognized that LSA-SVM is the most accurate classifier across seven datasets equal to 96.42 in “Australian Credit (Statelog)” except “Pima Indians diabetes” and “Spambase” datasets that is equal to 83.31% and 62.52% respectively.

**Figure 16:**
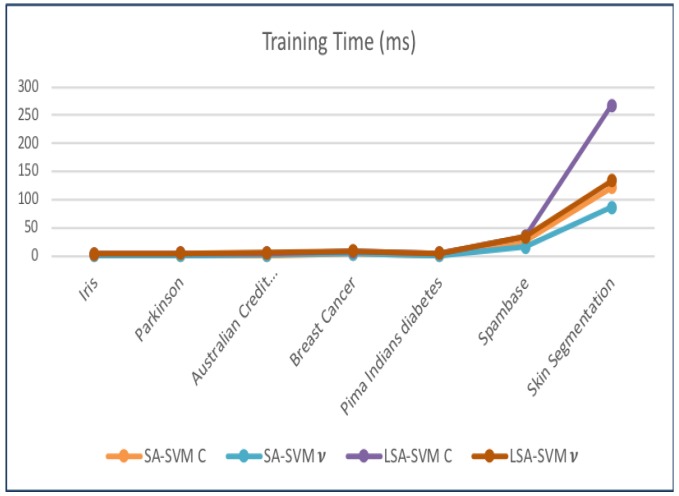
The training time consumption for each type of classifier.

The processing time (time consumption) of classifiers also calculated. Table 9 provides the training time. Figure 16 clarifies that the training time of LSA-SVM is one of the slowest classifiers, equated to other classifiers in overall datasets. Its performance worsens when vLSA-SVM works on big data like the “Skin Segmentation” dataset equal to 133.1 msec. The cLSA-SVM is the slowest classifier, taking around 267 ms to learn “Skin Segmentation” datasets while vSA-SVM shows the faster time, equal to 0.537 msec.

## 5. Conclusions

LSA-SVM has an advantage over SA-SVM as it works on label data instead of data value. In addition to the Myoelectric leg motion classification, LSA-SVM has been applied to a wide collection of classification problems using a UCI machine learning dataset. The experimental results show that LSA-SVM performs on a wide range of dataset sizes (small to large). Overall, LSA-SVM is a promising classifier for several classification applications, particularly for Myoelectric pattern recognition. In the training test, which was executed to compare the results of LSA-SVM with all other algorithms, the p-value correlated to the ANOVA test by a level of impact α = 0.05 was 0.038, which shows good statistical difference between these groups. Therefore, it can be concluded that LSA-SVM achieved better results than these algorithms. In addition, 68.80 percent sensitivity and 76.5 percent specificity was achieved to classify the testing data for the Hospital. Label Self-Advised Support Vector Machine (LSA-SVM) was implemented and projected the Self-Advised Support Vector Machine (SA-SVM) for leg motion recognition using sEMG signals. Overall, LSA-SVM could classify four leg movements with an accuracy of 99.06 percent, deeming it comparable with renowned classifiers such as SA-SVM, SVM. Therefore, LSA-SVM could improve the performance of the advised-based SVM.

This study presented a new Label classification method, called the Label Self-Advised-Support Vector Machine LSA-SVM, to diagnose leg movements for foot drop patients. Data were collected using a surface electromyography (sEMG) device from Foot Drop Patients from the Metro Rehabilitation Hospital in Sydney, Australia using Ethical Approval (UTS HREC NO. ETH15-0152). Also, the experimental results for the sEMG dataset, UCI and OpenSem benchmark datasets prove its assistance.

## Figures and Tables

**Table 1:** The hardware and software needed for the off-line application.

Component	Description	Picture
Hardware	Personal Computer Intel	
Hardware	EMG acquisition device, the FlexComp Infiniti™ System from Thought Technology with frequency sampling 2000 Hz	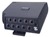
Hardware	Four EMG sensors: MyoScan™ T9503M Sensors from Though technology	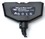
Hardware	Four electrodes	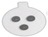
Hardware	OneProCom Infiniti USB Adapter—TT-USB	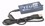
Hardware	One small piece of Fiber Optic Cable 15ft.—SA9480	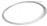
Hardware	OneProCom Infiniti USB Adapter—TT-USB	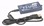
Software	Matlab R2016b	
Software	API library from Though Technology connecting the Flexcomp to Matlab	

**Table 2:** Characteristics for sick and healthy subjects collected for the hospital.

Gender	Age (Years)	Height (cm)	Weight (kg)	Min KFA(deg)	Speed (m/s)	*T* (Months)	BPL300
F	45	155.7	54.9	15	—	48	Yes
F	52	160	54.7	22	—	18	Yes
M	61	176	108	18	—	15	No
M	64	162	102	15	—	36	No
M	84	147	78.2	300	0.175	1	Yes
F	68	165	89.4	45	—	3	No
M	82	172	70	38	—	3	No
M	22	134.6	47	50	0.454	24	Yes
M	68	153	64.7	50	—	36	Yes
F	60	154.5	81	65	—	30	Yes
M	60	165	65	0	1.20		No
F	45	160	71	0	1.02		No
F	18	163	64	0	1.17		No

**Table 3:** Optimal parameters for all classifiers.

Dataset	C-SVM		*v*-SVM		Feat Type	Win. Size, Win. Inc.
	C	γ	C	γ		
Hospital	100	0.003	100	0.003	14	50, 15
crouch gait	100	0.003	100	0.003	14	20, 5

**Table 4:** The average classification accuracy of LSA-SVMacross two datasets using five-fold cross-validation compared with other classifiers.

Dataset	Accuracy (%)					
	SVM C	SVM *v*	SA-SVM C	SA-SVM *v*	LSA-SVM C	LSA-SVM *v*
Hospital	92.18	93.26	98.86	98.75	98.99	99.06
crouch gait	76.20	70.10	81.73	80.01	81.97	82.01

**Table 5:** The training time consumption for each type of classifier.

Dataset	Training Time (ms)					
	SVM C	SVM *v*	SA-SVM C	SA-SVM *v*	LSA-SVM C	LSA-SVM *v*
Hospital	55.3	54.3	107.9	46.3	70.7	69.4
crouch gait	48.5	55.1	36.3	38.0	48.9	48.4

**Table 6:** Data specification for benchmarking for 2-Classes datasets.

Dataset	Group	# Data	# Attributes
Iris	Small size	100	4
Parkinson		195	22
Australian Credit Approval (Statlog)	Medium size	689	4
Breast Cancer		699	9
Pima Indians diabetes		768	8
Spambase	Large size	4601	57
Skin Segmentation		245,057	3

**Table 7:** The optimal parameters used by each classifier in the UCI [19] dataset experiments for 2-classes.

Dataset	C-SVM		*v*-SVM		Feat Type	Win. Size, Win. Inc.
	C	γ	C	γ		
Iris	1	1	1	0.25	11	20, 5
Parkinson	300	0.125	3000	0.003	14	50, 15
Australian Credit Approval (Statlog)	0.5	0.125	214	0.003	14	20, 5
Breast Cancer	8200	0.003	214	0.003	13	50, 7
Spambase	3000	0.003	2050	0.005	14	500, 120
Pima Indians diabetes	8200	0.0078	2050	0.002	14	50, 15
Skin Segmentation	100	0.003	1	2	14	500, 150

**Table 8:** The accuracy of seven classifiers on various data using 5-fold cross validation for small and medium-size data and 3-fold cross validation for large size data for 2 classes.

Dataset	Accuracy (%)					
	SVM C	SVM *v*	SA-SVM C	SA-SVM *v*	LSA-SVM C	LSA-SVM *v*
Iris	92.85	90.12	85.71	85.84	92.85	92.86
Parkinson	72.32	70.0	71.43	64.29	71.43	74.10
Australian Credit (Statelog)	81.6	80.8	88.07	88.53	91.28	96.42
Breast Cancer	89.88	88.76	90.96	90.33	92.09	91.01
Pima Indians diabetes	82.5	80	78.4	77.5	80.95	78.3
Spambase	61.17	62.52	61.18	62.53	62.19	61.26
Skin Segmentation	83.31	72.26	60.50	67.47	84.23	84.43

**Table 9:** The training time consumption for each type of classifier.

Dataset	Training Time (ms)			
	SA-SVM C	SA-SVM *v*	LSA-SVM C	LSA-SVM *v*
Iris	0.837	0.701	3.6	4.1
Australian Credit (Statelog)	1.10	1.60	4.6	5.2
Breast Cancer	3.20	3.3	7.20	8.70
Pima Indians diabetes	0.901	0.836	5.0	4.7
Spambase	25.8	16.1	34.8	33.4
Skin Segmentation	121.4	86.3	267.2	133.1

## Abbreviations

The following abbreviations are used in this manuscript:

LAW	Lab Advised Weight
AW	Advised Weight
SVM	Support Vector Machine
FD	Foot drop
SA-SVM	Self Advised Support Vector Machine
LMN	Lower Motor Neuron
FES	Function Electrical Stimulation
M-PR	Myoelectric Pattern Recognition
SA-SVM	Self-Advised-Support Vector Machine
LSA-SVM	Label Self-Advised-Support Vector Machine
LCM	label classification methods
RBF	Radial Basis Function
MD	Misclassify Datasets
EMG	Electromyography
sEMG	surface EMG
TA	Tibias Anterior
RF	Rectus Femurs
Gas	Gastrocnemius
Sol	Soule
TA	Tibias Anterior
CG	Crouch Gait
LDA	Linear discriminant analysis
TD-AR	Timing Domain-Autoregressive
SCC	single class classification
SLC	Single Label Classification
MDPI	Multidisciplinary Digital Publishing Institute
DOAJ	Directory of open access journals
TLA	Three letter acronym
LD	linear dichroism

## References

[1] Westhout F.D., Paré L.S., Linskey M.E. (2007). Central causes of foot drop: Rare and underappreciated differential diagnoses. J. Spinal Cord Med..

[2] Hiam D.S. (2017). The Gale Encyclopedia of Neurological Disorders.

[3] Gastounioti A., Makrodimitris S., Golemati S., Kadoglou N.P., Liapis C.D., Nikita K.S. (2015). A novel computerized tool to stratify risk in carotid atherosclerosis using kinematic features of the arterial wall. IEEE J. Biomed. Health Inform..

[4] Vapnik V., Vapnik V. (1998). Statistical Learning Theory.

[5] Wang Z., Xue X., Ma Y., Guo G. (2014). Multi-Class Support Vector Machine. Support Vector Machines Applications.

[6] Masood A., Al-Jumaily A. (2015). SA-SVM based automated diagnostic system for skin cancer. Proc. SPIE.

[7] Maali Y., Al-Jumaily A. (2013). Self-advising support vector machine. Knowl.-Based Syst..

[8] Masood A., Al-Jumaily A., Anam K. (2014). Texture analysis based automated decision support system for classification of skin cancer using SA-SVM. Proceedings of the International Conference on Neural Information Processing.

[9] Mohammed A.A., Sajjanhar A. Robust single-label classification of facial attributes. Proceedings of the 2017 IEEE International Conference on Multimedia & Expo Workshops (ICMEW).

[10] Xu J.W., Suzuki K. (2014). Max-AUC feature selection in computer-aided detection of polyps in CT colonography. IEEE J. Biomed. Health Inform..

[11] Herrera F., Charte F., Rivera A.J., Del Jesus M.J. (2016). Multilabel classification. Multilabel Classification.

[12] Masood A. (2016). Developing Improved Algorithms for Detection and Analysis of Skin Cancer. Ph.D. Thesis.

[13] Anam K., Al Jumaily A., Maali Y. (2014). Index Finger Motion Recognition using self-advise support vector machine. Int. J. Smart Sens. Intell. Syst..

[14] Masood A., Al-Jumaily A., Anam K. Self-supervised learning model for skin cancer diagnosis. Proceedings of the 7th International IEEE/EMBS Conference on Neural Engineering (NER).

[15] Kolesov A., Kamyshenkov D., Litovchenko M., Smekalova E., Golovizin A., Zhavoronkov A. (2014). On multilabel classification methods of incompletely labeled biomedical text data. Comput. Math. Methods Med..

[16] Read J., Pfahringer B., Holmes G., Frank E. (2011). Classifier chains for multi-label classification. Mach. Learn..

[17] Reynolds J.S., Goldsmith W.T., Day J.B., Abaza A.A., Mahmoud A.M., Afshari A.A., Barkley J.B., Petsonk E.L., Kashon M.L., Frazer D.G. (2016). Classification of voluntary cough airflow patterns for prediction of abnormal spirometry. IEEE J. Biomed. Health Inform..

[18] Sakai H., Liu C., Nakata M. Information Dilution: Granule-Based Information Hiding in Table Data—A Case of Lenses Data Set in UCI Machine Learning Repository. Proceedings of the 2016 Third International Conference on Computing Measurement Control and Sensor Network (CMCSN).

[19] Dua D., Graff C. (2017). UCI Machine Learning Repository.

[20] Ashok P., Nawaz G.K. Detecting outliers on UCI repository datasets by Adaptive Rough Fuzzy clustering method. Proceedings of the 2016 Online International Conference on Green Engineering and Technologies (IC-GET).

[21] Trejo R.L., Vázquez J.P.G., Ramirez M.L.G., Corral L.E.V., Marquez I.R. Hand goniometric measurements using leap motion. Proceedings of the 2017 14th IEEE Annual Consumer Communications & Networking Conference (CCNC).

[22] Osojnik A., Panov P., Džeroski S. (2017). Multi-label classification via multi-target regression on data streams. Mach. Learn..

[23] Ren D., Ma L., Zhang Y., Sunderraman R., Fox P.T., Laird A.R., Turner J.A., Turner M.D. Online biomedical publication classification using multi-instance multi-label algorithms with feature reduction. Proceedings of the IEEE 14th International Conference on Cognitive Informatics & Cognitive Computing (ICCI*CC).

